# GPSM2 Promotes Pancreatic Cancer Progression Through METTL3‐Mediated m6A Modification of YAP1 mRNA

**DOI:** 10.1111/jcmm.71224

**Published:** 2026-06-02

**Authors:** Jiajun Xiu, Li Qiao, Miaomiao Li, Xiaoan Hu, Zixuan Shen, Rui Yang, Hairu Zhang, Zhe Dong, Xuelei Liu, Yinghui Zhang

**Affiliations:** ^1^ Oujiang Laboratory (Zhejiang Lab for Regenerative Medicine, Vision and Brain Health) Wenzhou Medical University Wenzhou Zhejiang China; ^2^ Department of Laboratory Medical Center General Hospital of Northern Theater Command Shenyang People's Republic of China; ^3^ Medicine Laboratory Centre The First Hospital of Lanzhou University Lanzhou People's Republic of China; ^4^ Center of Reproductive Medicine Shenyang Women's and Children's Hospital Shenyang People's Republic of China; ^5^ Department of Plastic Surgery The First Affiliated Hospital of Jinan University Guangzhou Guangdong China; ^6^ Department of Pathology General Hospital of Northern Theater Command Shenyang People's Republic of China; ^7^ Department of Pathology Shengjing Hospital, China Medical University Shenyang People's Republic of China

**Keywords:** GPSM2, IGF2BP2/3, m6A, METTL3, pancreatic cancer, YAP1

## Abstract

Pancreatic cancer poses a major therapeutic challenge due to its insidious onset and difficulty in early diagnosis. G‐protein signalling modulator 2 (GPSM2), a member of the G‐protein signalling regulator family, is highly expressed in various tumour tissues; however, its role in pancreatic cancer remains largely undefined. Yes‐associated protein 1 (YAP1), a transcriptional co‐activator, has been recognised as a central node in the growth‐promoting signalling pathways of pancreatic cancer. Nevertheless, whether GPSM2 contributes to pancreatic cancer progression through the regulation of YAP1 has not yet been elucidated. In this study, transcriptome analysis of 183 pancreatic cancer patients from The Cancer Genome Atlas (TCGA) dataset identified GPSM2 as a survival‐associated gene in pancreatic cancer. Functionally, we demonstrated that GPSM2 promotes colony formation and invasion of pancreatic cancer cells and was found to be mechanistically linked to the regulation of YAP1. Molecular investigations revealed that GPSM2 significantly upregulated YAP1 mRNA levels. Further analysis demonstrated that GPSM2 enhanced the N6‐methyladenosine (m6A) modification of YAP1 mRNA in a METTL3‐dependent manner. The KH3‐4 domain of the m6A reader proteins IGF2BP2 and IGF2BP3 specifically recognised the m6A‐modified YAP1 transcripts, thereby stabilising YAP1 mRNA and increasing YAP1 protein expression, which in turn promoted colony formation and invasion in pancreatic cancer cells. These findings provide novel insights into the molecular mechanisms underlying pancreatic cancer progression and may offer promising therapeutic targets for future intervention.

**Abbreviations**
GPSM2: G‐protein signalling modulator 2
IGF2BP: Insulin; like growth factor 2 mRNA‐binding protein
METTL14: Methyltransferase‐like 14
METTL3: Methyltransferase‐like 3
PDAC: Pancreatic ductal adenocarcinoma
TAZ: Transcriptional co‐activator with PDZ‐binding motif
VP: Verteporfin
WTAP: Wilms tumour 1 associated protein
YAP1: Yes‐associated protein 1

## 1 Background

Pancreatic cancer is often referred to as the ‘king of cancers’. The majority of pancreatic cancers are classified as pancreatic ductal adenocarcinoma (PDAC), which is characterised by high aggressiveness, late‐stage diagnosis and poor prognosis, with a 5‐year survival rate of only 13% [1, 2]. Due to its insidious onset and the difficulty of early detection, more than 80% of PDAC patients are diagnosed at an advanced stage, with distant metastases or significant vascular invasion, rendering them unsuitable for surgical resection. Only approximately 20% of patients are candidates for potentially curative surgery [3, 4]. Despite significant advances in cancer research, PDAC remains one of the most chemoresistant malignancies, contributing to its persistently high mortality rate. Therefore, there is an urgent need to explore novel therapeutic strategies to improve the survival outcomes of patients with PDAC.

G‐protein signalling modulator 2 (GPSM2) is a member of the G‐protein signalling regulator family and functions as a non‐receptor‐dependent protein that modulates G‐protein activity. It plays a critical role in mitotic spindle positioning and cell cycle regulation [5]. The GPSM2 protein consists of 684 amino acids and comprises eight N‐terminal tetratricopeptide repeats (TPRs) and four C‐terminal GoLoco motifs [6]. GPSM2 has been implicated in various types of cancer. For instance, it is involved in the epithelial–mesenchymal transition (EMT) process in non‐small cell lung cancer, and its silencing in breast cancer leads to defective cell division and significant inhibition of cancer cell proliferation [7, 8]. However, the functional role and underlying mechanisms of GPSM2 in pancreatic cancer remain largely unknown.

N6‐methyladenosine (m6A) methylation is the most prevalent epigenetic modification in RNA and is closely associated with cellular proliferation, metastasis and metabolic reprogramming in a wide range of cancers, including pancreatic cancer [9, 10, 11]. M6A methylation plays a pivotal role in post‐transcriptional RNA regulation, particularly by modulating RNA structural stability and expression levels [12, 13].

YAP1, a transcriptional co‐activator, is a key component of the Hippo/YAP signalling pathway and plays a crucial role in promoting tumour initiation and progression [14, 15, 16]. Elevated expression of YAP1 has been reported in various types of cancer, including pancreatic cancer. For instance, Ren et al. demonstrated that silencing or inhibiting sodium‐glucose cotransporter 2 (SGLT2) suppressed the activation of Hippo signalling by downregulating YAP1 expression [17]. Moreover, RNA polymerase II‐associated factor 1 (PAF1) has been shown to cooperate with YAP1 during acinar‐to‐ductal metaplasia (ADM) and pancreatic cancer development, while verteporfin inhibits ADM and pancreatic cancer cell growth by targeting the PAF1/YAP1/SOX9 axis in both in vitro and ex vivo models [18]. However, whether GPSM2 contributes to pancreatic cancer progression by regulating YAP1 remains unknown. In this study, we explored the regulatory mechanisms of GPSM2 and YAP1 in pancreatic cancer. We first found that GPSM2 promotes colony formation and invasion of pancreatic cancer cells. Mechanistically, GPSM2 was shown to recognise the m6A modification of YAP1 mRNA through the m6A reader proteins IGF2BP2 and IGF2BP3. Collectively, these findings identify GPSM2 as a potential survival‐associated biomarker and therapeutic target in pancreatic cancer.

## 2 Results

### 2.1 Integrated Transcriptomic Analysis Identifies GPSM2 as a Survival‐Associated Gene in Pancreatic Cancer

**FIGURE 1:**
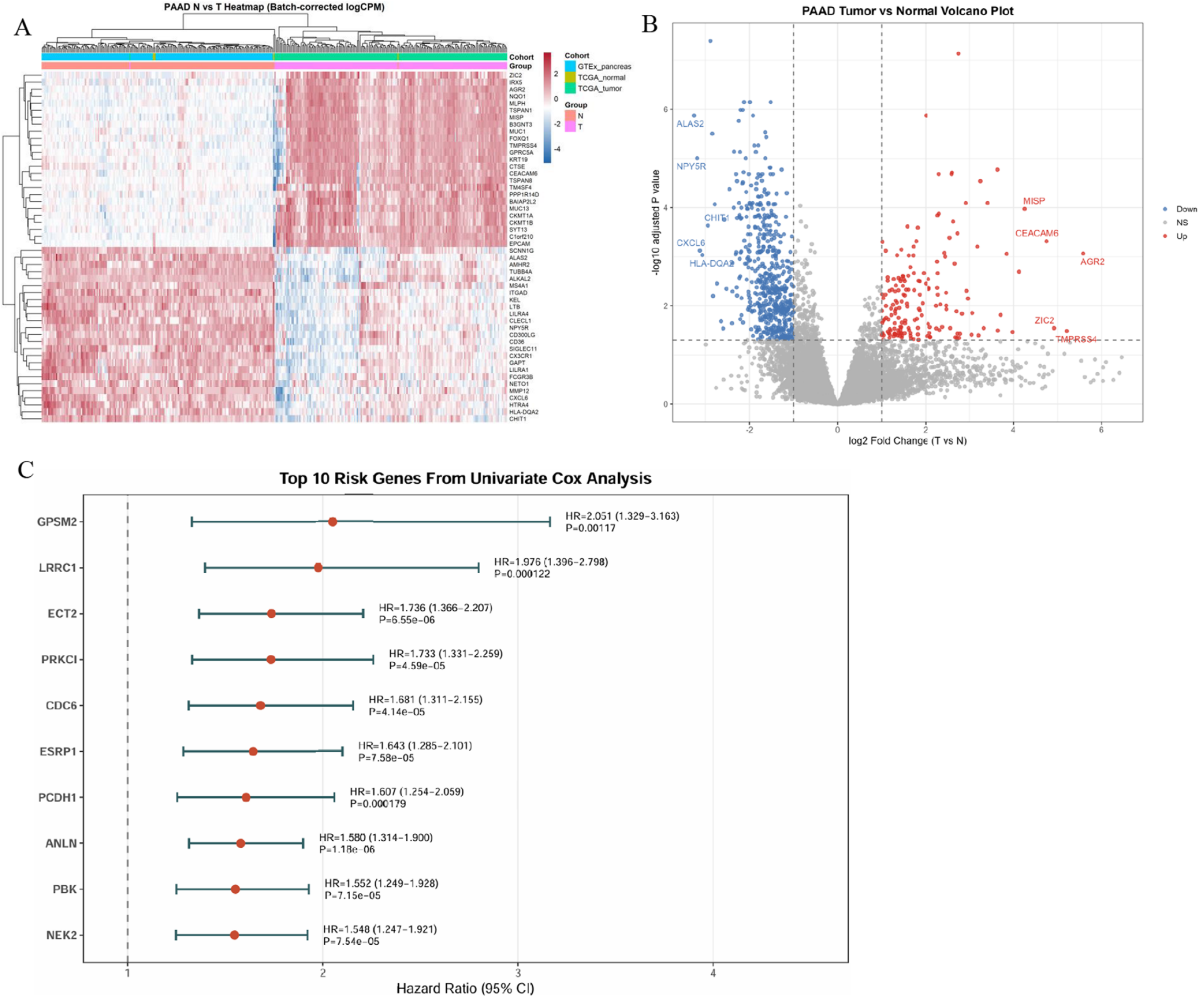
Integrated transcriptomic analysis of pancreatic cancer identifies GPSM2 as a survival‐associated gene. (A) Heatmap of differentially expressed genes (DEGs) between pancreatic cancer tissues and normal pancreatic tissues after integration of TCGA‐PAAD and GTEx datasets and batch correction. (B) Volcano plot showing significantly upregulated and downregulated genes in pancreatic cancer compared with normal pancreatic tissues. (C) Forest plot showing the top 10 survival‐associated genes identified by univariate Cox regression analysis.

To improve the robustness of the tumour–normal differential expression analysis, we integrated transcriptomic data from TCGA‐PAAD tumour samples with normal pancreatic tissue samples from GTEx, together with the available adjacent normal pancreatic samples from TCGA, followed by normalisation and batch correction. Using the predefined screening criteria, we identified a set of differentially expressed genes (DEGs) between pancreatic cancer and normal pancreatic tissues. The heatmap showed a clear separation between tumour and normal samples and the volcano plot further illustrated the distribution of significantly upregulated and downregulated genes in pancreatic cancer (Figure 1A,B). We next evaluated the survival relevance of the DEGs by univariate Cox regression analysis. Among the top survival‐associated genes, GPSM2 ranked as a prominent risk‐associated gene, with a hazard ratio (HR) of 2.051 (95% CI:1.329–3.163, *p* = 0.001177) (Figure 1E). These findings suggest that GPSM2 is significantly associated with unfavourable overall survival in pancreatic cancer.

### 2.2 GPSM2 Is Upregulated in Pancreatic Cancer and Associated With Poor Overall Survival

**FIGURE 2:**
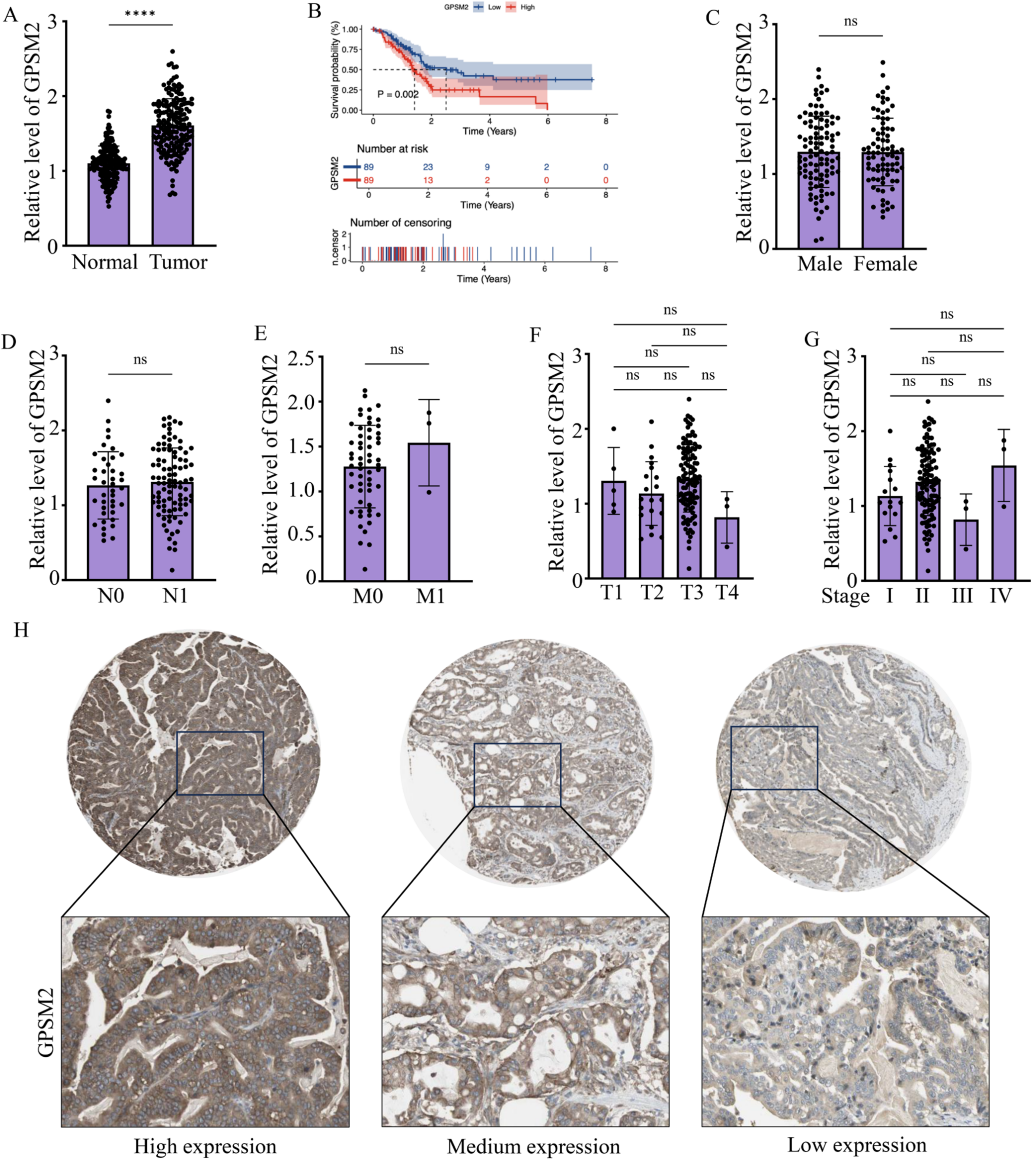
Expression of GPSM2 in samples and its correlation with survival and clinicopathological staging in pancreatic cancer patients. (A) Differential expression of GPSM2 between normal and tumour tissues. *p < 0.05. (B) Kaplan–Meier survival analysis of GPSM2 Expression performed using the R packages survival and survminer. (C) Comparison of GPSM2 Expression between male and female patients. ns, not significant (*p* > 0.05). (D–G) Correlation analysis of GPSM2 expression with clinicopathological staging parameters. ns, not significant (*p* > 0.05). (H) Immunohistochemistry (IHC) data from the Human Protein Atlas showing representative images of high, medium and low GPSM2 Expression in pancreatic cancer tissues.

We next examined GPSM2 expression in the integrated tumour–normal cohort. Compared with normal pancreatic tissues, GPSM2 expression was significantly elevated in pancreatic cancer tissues (Figure 2A). Kaplan–Meier survival analysis further demonstrated that patients with high GPSM2 expression had significantly shorter overall survival than those with low GPSM2 expression (Figure 2B). We then analysed the association between GPSM2 expression and clinicopathological characteristics in the TCGA cohort. No significant differences in GPSM2 expression were observed with respect to sex, N stage, M stage, T stage or overall pathological stage (Figure 2C–G). To further evaluate GPSM2 protein expression in pancreatic cancer tissues, we examined immunohistochemical images from the Human Protein Atlas, which showed representative cases with high, medium and low GPSM2 expression (Figure 2H). Collectively, these data indicate that GPSM2 is aberrantly upregulated in pancreatic cancer and is associated with poor survival, although its expression was not significantly correlated with the available clinicopathological staging variables in this cohort.

### 2.3 GPSM2 Promotes the Proliferation and Invasion of Pancreatic Cancer Cells

**FIGURE 3:**
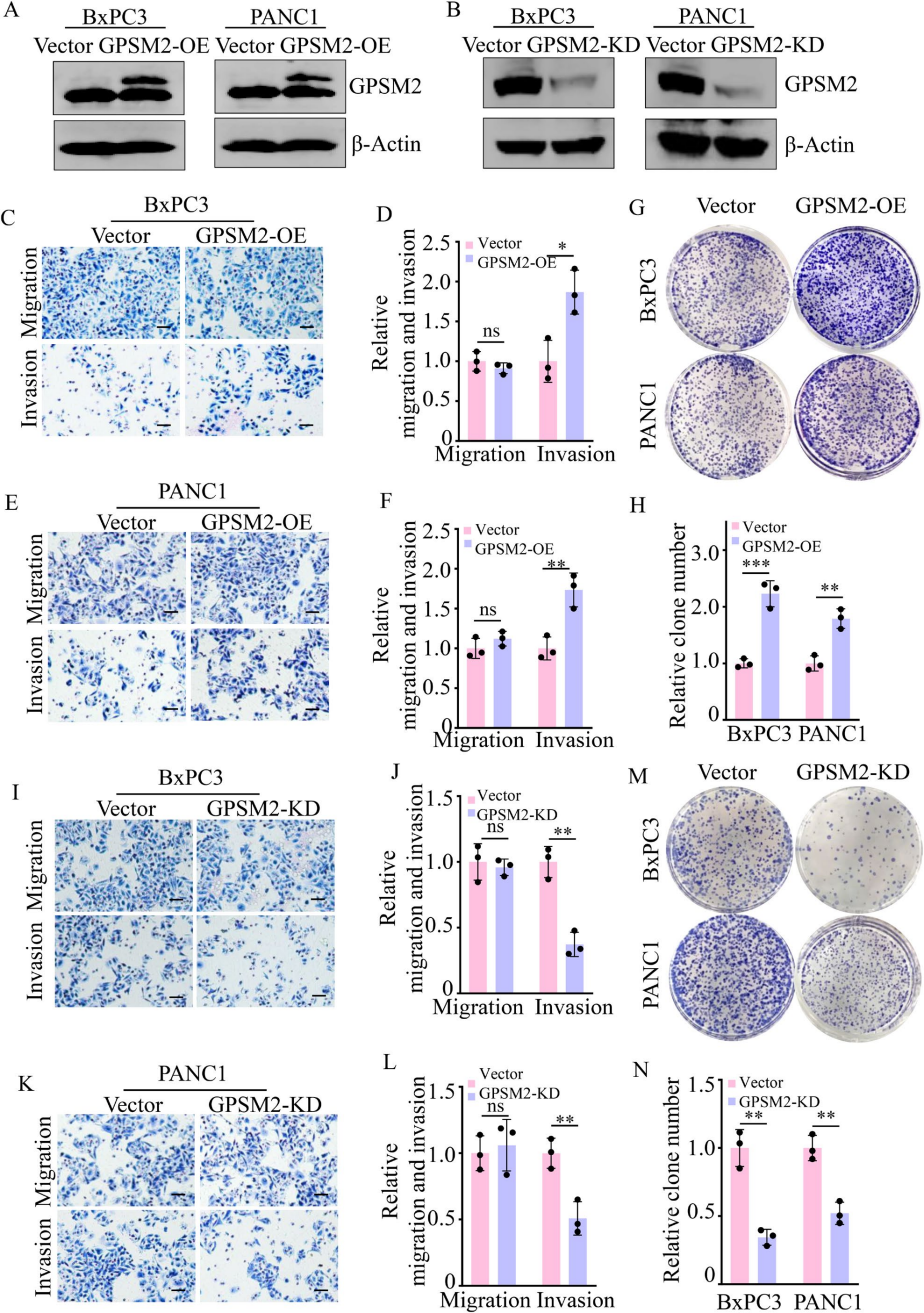
GPSM2 promotes the malignant progression of pancreatic cancer cells. (A) Western blot analysis confirming overexpression of GPSM2 protein in BxPC‐3 and PANC‐1 cells. (B) Western blot analysis confirming knockdown of GPSM2 protein in BxPC‐3 and PANC‐1 cells. (C‐D) Transwell assays evaluating the effects of GPSM2 overexpression on migration and invasion of BxPC‐3 cells. Statistical analyses are shown; ns, not significant (*p* > 0.05); *p < 0.05. (E‐F) Transwell assays evaluating the effects of GPSM2 overexpression on migration and invasion of PANC‐1 cells. Statistical analyses are shown; ns, not significant (*p* > 0.05); **p < 0.01. (G‐H) Colony formation assays demonstrating the proliferative effect of GPSM2 overexpression in BxPC‐3 and PANC‐1 cells. Colonies were counted 14 days post‐seeding. **p < 0.01, ***p < 0.001. (I‐J) Transwell assays assessing the effects of GPSM2 knockdown on migration and invasion of BxPC‐3 cells. Statistical analyses are shown; ns, not significant (*p* > 0.05); **p < 0.01. (K‐L) Transwell assays assessing the effects of GPSM2 knockdown on migration and invasion of PANC‐1 cells. Statistical analyses are shown; ns, not significant (*p* > 0.05); *p < 0.05. (M‐N) Colony formation assays showing the inhibitory effect of GPSM2 knockdown on BxPC‐3 and PANC‐1 cell proliferation. Colonies were counted 14 days post‐seeding. **p < 0.01.

To investigate the biological function of GPSM2 in pancreatic cancer, we established stable cell lines by overexpressing or silencing GPSM2 in two pancreatic cancer cell lines, BxPC‐3 and PANC‐1. The efficiency of GPSM2 modulation is shown in Figure 3A,B. Overexpression of GPSM2 significantly enhanced the invasive capacity of both BxPC‐3 and PANC‐1 cells (Figure 3C–F), and markedly promoted cell proliferation in both cell lines (Figure 3G,H). In contrast, knockdown of GPSM2 significantly suppressed the invasion ability of BxPC‐3 and PANC‐1 cells (Figure 3I–L), and notably reduced their proliferative capacity (Figure 3M,N).

### 2.4 GPSM2 Facilitates Pancreatic Cancer Cell Colony Formation and Invasion via YAP1

**FIGURE 4:**
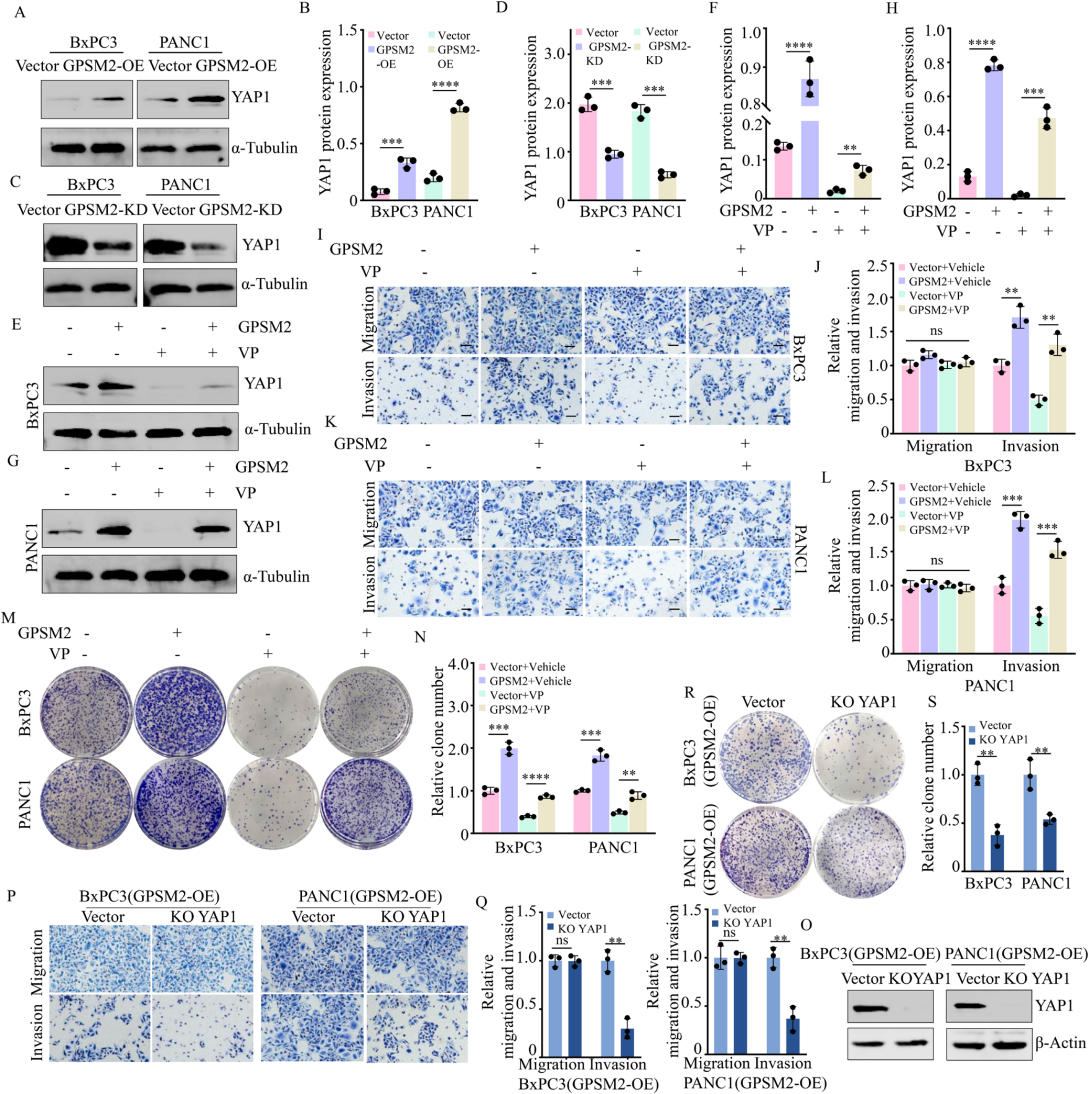
GPSM2 promotes colony formation and invasion of pancreatic cancer cells through YAP1. (A‐B) Western blot analysis of YAP1 protein expression in BxPC‐3 and PANC‐1 cells following GPSM2 overexpression (GPSM2‐OE). ***p < 0.001, ****p < 0.0001. (C‐D) Western blot analysis of YAP1 protein expression in BxPC‐3 and PANC‐1 cells following GPSM2 knockdown (GPSM2‐KD). ***p < 0.001. (E‐F) Western blot analysis of YAP1 expression in BxPC‐3 cells treated with Verteporfin (a YAP1 inhibitor, 2.5 μg/mL) after GPSM2 overexpression. **p < 0.01, ****p < 0.0001. (G‐H) Western blot analysis of YAP1 expression in PANC‐1 cells treated with Verteporfin after GPSM2 overexpression. ***p < 0.001, ****p < 0.0001. (I‐J) Transwell assays assessing the migration and invasion of BxPC‐3 cells treated with Verteporfin following GPSM2 overexpression. Statistical analyses are shown; ns, not significant (*p* > 0.05); **p < 0.01. (K‐L) Transwell assays assessing the migration and invasion of PANC‐1 cells treated with Verteporfin following GPSM2 overexpression. Statistical analyses are shown; ns, not significant (*p* > 0.05); ***p < 0.001. (M‐N) Colony formation assays demonstrating the effect of Verteporfin treatment on the proliferative capacity of BxPC‐3 and PANC‐1 cells after GPSM2 overexpression. Colonies were counted 14 days after seeding. **p < 0.01, ***p < 0.001, ****p < 0.0001. (O) Western blot analysis of YAP1 protein expression in BxPC‐3 and PANC‐1 cells following GPSM2 overexpression (GPSM2‐OE). (P‐Q) Transwell assays assessing the migration and invasion of BxPC‐3 and PANC‐1 cells overexpressing GPSM2 following YAP1 knockout (KO YAP1). Statistical analyses are shown; ns, not significant (*p* > 0.05); **p < 0.01. (R‐S) Colony formation assays showing the inhibitory effect of BxPC‐3 and PANC‐1 cells overexpressing GPSM2 following YAP1 knockout. Colonies were counted 14 days post‐seeding. **p < 0.01.

To investigate whether GPSM2 regulates YAP1 protein expression to promote the cell colony formation and metastasis of pancreatic cancer cells, We performed Western blot analysis to examine YAP1 expression following GPSM2 overexpression or knockdown. The results demonstrated that GPSM2 overexpression significantly upregulated YAP1 protein levels (Figure 4A,B), While GPSM2 knockdown markedly suppressed YAP1 protein expression (Figure 4C,D). Moreover, treatment with the YAP1 inhibitor Verteporfin (VP) further enhanced the effect of GPSM2 on promoting YAP1 protein expression in BxPC‐3 and PANC‐1 cells (Figure 4E–H). In parallel, GPSM2 notably increased the invasive and proliferative capacities of BxPC‐3 and PANC‐1 cells (Figure 4I–N). To further demonstrate that GPSM2 regulates colony formation and invasion of pancreatic cancer cells through YAP1, we constructed stable YAP1 knockout cell lines within GPSM2‐overexpressing cells (Figure 4O). The results showed that the ability of GPSM2 to promote pancreatic cancer cell metastasis was markedly attenuated (Figure 4P,Q), and its effect on enhancing colony formation was also significantly reduced (Figure 4R,S). These results demonstrate that GPSM2 facilitates pancreatic cancer cell colony. formation and invasion via YAP1.

### 2.5 GPSM2 Enhances YAP1 mRNA Levels via m6A Modification

**FIGURE 5:**
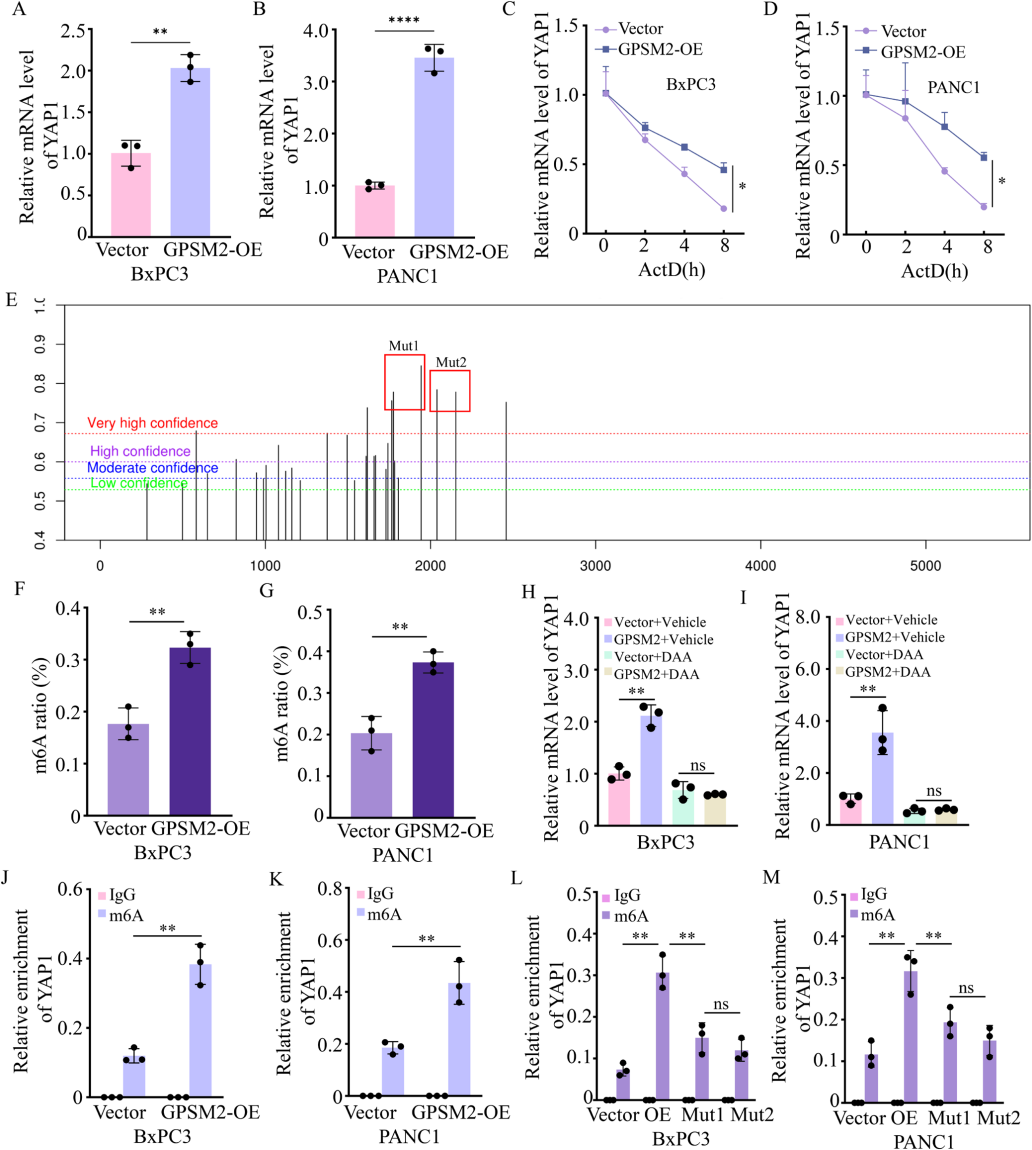
GPSM2 enhances YAP1 mRNA levels via m6A modification. (A‐B) RT‐qPCR analysis of YAP1 mRNA levels following GPSM2 overexpression (GPSM2‐OE) in BxPC‐3 and PANC‐1 cells. **p < 0.01, ****p < 0.0001. (C‐D) RT‐qPCR analysis of YAP1 mRNA stability after GPSM2 overexpression (GPSM2‐OE) in BxPC‐3 and PANC‐1 cells. *p < 0.05. (E) Bioinformatic prediction of putative m^6^A modification sites on YAP1 mRNA using SRAMP databases. (F‐G) mRNA m6A levels were detected using EpiQuik m6A RNA Methylation Quantification Kit following GPSM2 overexpression (GPSM2‐OE) in BxPC‐3 and PANC‐1 cells. **p < 0.01. (H‐I) Relative YAP1 mRNA levels in BxPC‐3 and PANC‐1 cells treated with the methylation inhibitor 3‐deazaadenosine (DAA, 200 μM). ns, not significant (*p* > 0.05), **p < 0.01. (J‐K) Validation of m6A methylation on YAP1 mRNA in BxPC‐3 and PANC‐1 cells by MeRIP‐qPCR. **p < 0.01. (L‐M) For mutant 1 and 2 reporter plasmids, cytosine (C) replaced marked adenosine (A) in m6A motif, examine the specific modifications of YAP1.

To further investigate the mechanism by which GPSM2 regulates YAP1, we assessed the mRNA levels of YAP1. The results demonstrated that GPSM2 markedly increased YAP1 mRNA levels in both BxPC‐3 and PANC‐1 cells (Figure 5A,B). Further analysis of mRNA decay kinetics revealed that GPSM2 significantly enhanced the stability of YAP1 mRNA (Figure 5C,D). N6‐methyladenosine (m6A) is the most abundant internal modification in eukaryotic mRNA [19]. M6A indirectly affects RNA processing by recruiting specific reader proteins, some of which function as regulators of mRNA stability [20, 21]. To explore whether GPSM2 promotes YAP1 mRNA stability through m6A modification, the online tool SRAMP (http://www.cuilab.cn/sramp) was used to predict potential m6A modification sites, and two mutant plasmids (Mut1 and Mut2) were constructed to investigate the specific m6A modification sites on YAP1 (Figure 5E). Following GPSM2 overexpression, m6A enrichment on YAP1 mRNA was markedly increased (Figure 5F,G). Treatment of pancreatic cancer cells with the methylation inhibitor 3‐deazaadenosine (DAA) significantly abrogated the GPSM2‐induced increase in YAP1 mRNA levels (Figure 5H,I). To further confirm that GPSM2 promotes YAP1 mRNA levels via m6A methylation, we performed methylated RNA immunoprecipitation followed by qPCR (MeRIP‐qPCR). The results showed a significant enrichment of m6A‐modified YAP1 transcripts using an m6A‐specific antibody compared to the IgG control (Figure 5J,K). Furthermore, in the mutant 1 and mutant 2 reporter plasmids, the adenosine (A) residues within the predicted m6A motifs were substituted with cytosine (C). As shown in Figure 5L,M, both Mut1 and Mut2 significantly reduced the enrichment of YAP1 mRNA. Collectively, these findings indicate that GPSM2 enhances YAP1 mRNA expression by promoting m6A methylation.

### 2.6 METTL3 Mediates the m6A Modification of YAP1 mRNA

**FIGURE 6:**
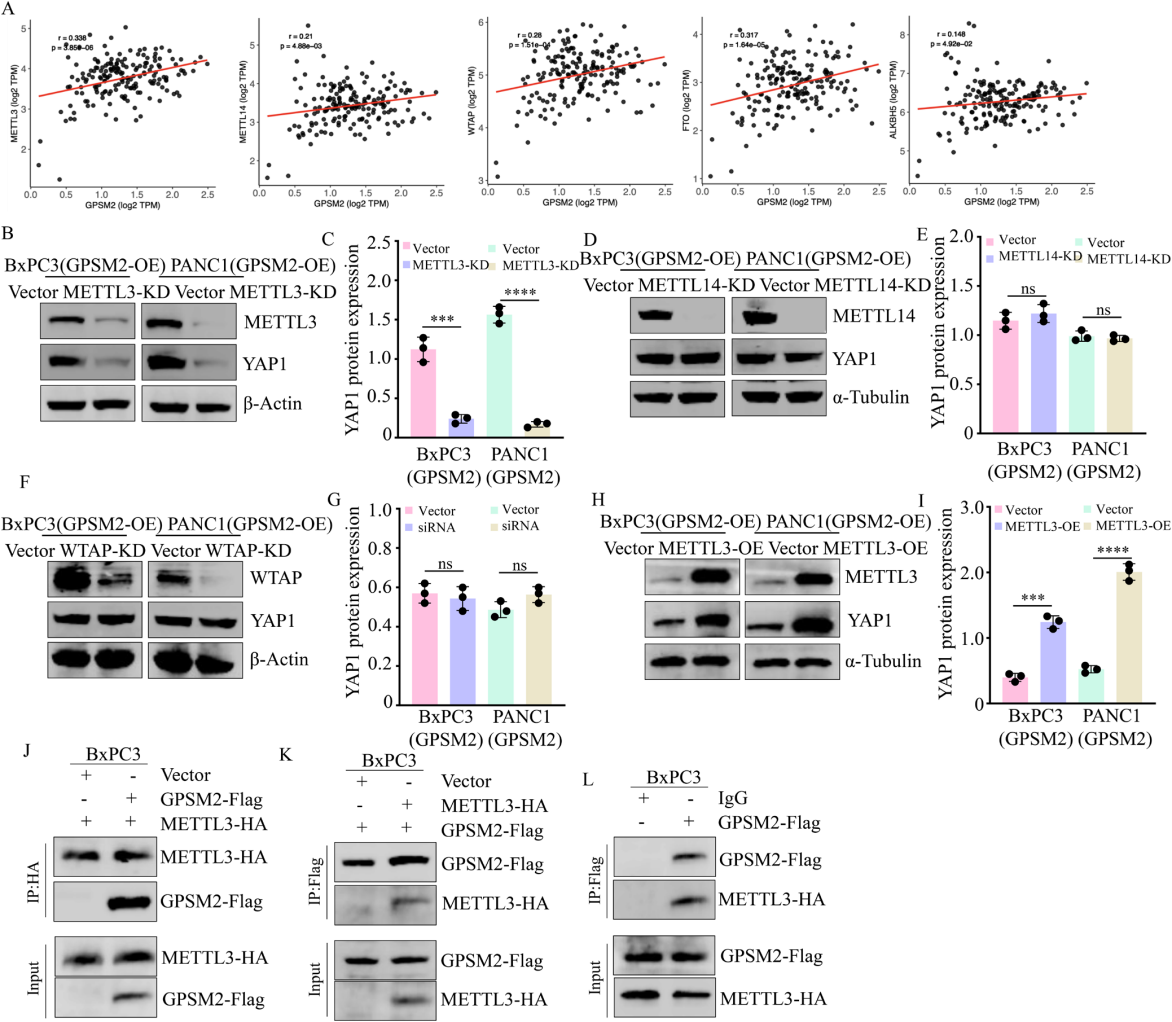
GPSM2 regulates the m6A modification of YAP1 mRNA via METTL3. (A) Correlation analysis between GPSM2 and key m6A regulators (METTL3, METTL14, WTAP, FTO and ALKBH5) using TCGA datasets. Analyses were performed with R packages *ggplot2* and *ggpubr*. (B‐C) Western blot analysis of METTL3 expression in BxPC‐3 (GPSM2‐OE) and PANC‐1 (GPSM2‐OE) cells, and its impact on YAP1 protein levels. ***p < 0.001, ****p < 0.0001. (D‐E) Western blot analysis of METTL14 expression in BxPC‐3 (GPSM2‐OE) and PANC‐1 (GPSM2‐OE) cells, and its effect on YAP1 protein expression. ns, not significant (*p* > 0.05). (F‐G) Western blot analysis of WTAP expression in BxPC‐3 (GPSM2‐OE) and PANC‐1 (GPSM2‐OE) cells, and its effect on YAP1 protein expression. ns, not significant (*p* > 0.05). (H‐I) Western blot analysis of METTL3 knockdown efficiency in BxPC‐3 (GPSM2‐OE) and PANC‐1 (GPSM2‐OE) cells and its effect on YAP1 protein levels. ***p < 0.001, ****p < 0.0001. (J‐K) Immunoprecipitation was performed using Anti‐HA‐tag mAb‐Magnetic Beads (J) or Anti‐DDDDK‐tag mAb‐Magnetic Beads (K) to detect the interaction between GPSM2 and METTL3. (L) BxPC3 cell lysates were subject to immunoprecipitation with control IgG and anti‐GPSM2 antibodies.

M6A modification is dynamically regulated by the coordinated actions of methyltransferases and demethylases [22]. To investigate the potential regulatory enzymes involved in YAP1 m6A modification, we analysed the correlation between GPSM2 and key m6A regulators using data from the TCGA pancreatic cancer cohort. GPSM2 expression was found to be positively correlated with both methyltransferases (METTL3, METTL14, WTAP) and demethylases (FTO, ALKBH5) (Figure 6A). However, given that global m6A levels are elevated in pancreatic cancer [23]. we excluded the potential involvement of demethylases FTO and ALKBH5. We then knocked down METTL3, METTL14 or WTAP in GPSM2‐overexpressing pancreatic cancer cell lines and evaluated YAP1 expression (Figure 6B,D,F). Notably, silencing METTL14 or WTAP did not affect GPSM2‐induced YAP1 expression (Figure 6D,G). In contrast, METTL3 knockdown abolished the upregulation of YAP1 protein levels by GPSM2 (Figure 6B,C). These findings suggest that METTL3 is the primary methyltransferase responsible for GPSM2‐mediated m6A modification of YAP1 mRNA. To further validate this hypothesis, we ectopically overexpressed METTL3 in two pancreatic cancer cell lines (Figure 6H). The results showed that METTL3 overexpression significantly enhanced the ability of GPSM2 to promote YAP1 expression (Figure 6H,I). To further validate the potential regulatory mechanism between GPSM2 and METTL3, we co‐transfected GPSM2‐Flag and METTL3‐HA into BxPC‐3 cells, followed by assessment of protein–protein interactions using co‐immunoprecipitation assays. The results demonstrated that GPSM2 interacted with METTL3 upon immunoprecipitation with either HA‐ or Flag antibody–conjugated magnetic beads (Figure 6J,K). Importantly, an interaction between endogenous GPSM2 and METTL3 was also detected in BxPC‐3 cells (Figure 6L). Collectively, these findings indicate that GPSM2 regulates m6A modification of YAP1 mRNA through its interaction with METTL3.

### 2.7 GPSM2 Enhances YAP1 mRNA Stability via the KH3‐4 Domains of IGF2BP2 and IGF2BP3

**FIGURE 7:**
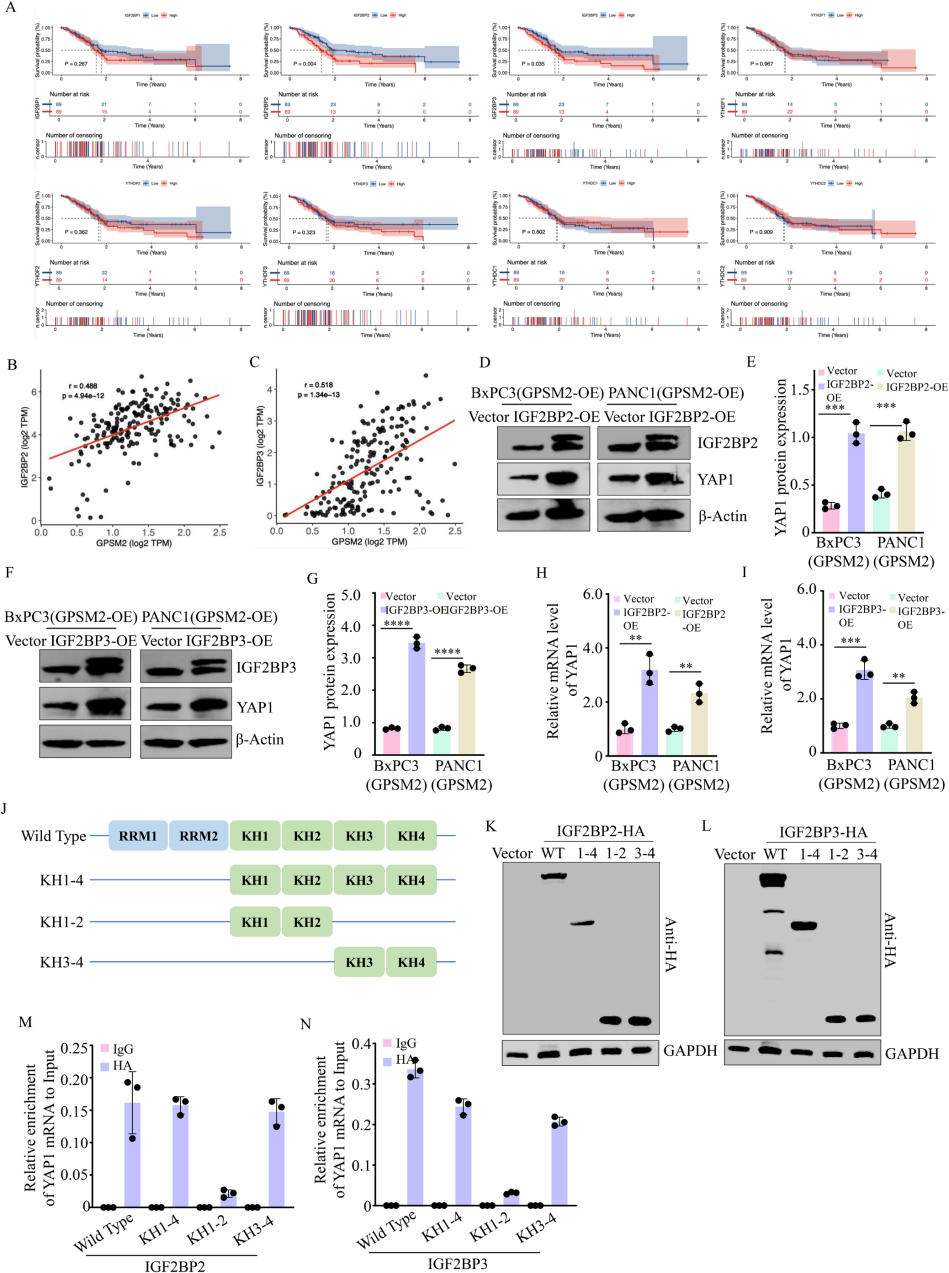
The KH3‐4 domains of IGF2BP2 and IGF2BP3 promote YAP1 mRNA stability. (A) Kaplan–Meier survival analysis performed using the R packages survival and survminer. (B‐C) Correlation analyses between GPSM2 and IGF2BP2 (B) or IGF2BP3 (C) conducted using R packages ggplot2 and ggpubr. (D‐E) Western blot analysis verifying IGF2BP2 overexpression efficiency and its effect on YAP1 protein levels in BxPC‐3(GPSM2‐OE) and PANC‐1(GPSM2‐OE) cells. ***p < 0.001. (F‐G) Western blot analysis verifying IGF2BP3 overexpression efficiency and its effect on YAP1 protein levels in BxPC‐3(GPSM2‐OE) and PANC‐1(GPSM2‐OE) cells. ****p < 0.0001. (H‐I) RT‐qPCR analysis of YAP1 mRNA levels following IGF2BP2 overexpression in BxPC‐3(GPSM2‐OE) and PANC‐1(GPSM2‐OE) cells. **p < 0.01, ***p < 0.001. (J) Schematic representation of wild‐type (WT) and three KH domain‐deleted plasmids tagged with HA for IGF2BP2 and IGF2BP3. (K‐L) Western blot analysis showing the expression efficiency of IGF2BP2/3 WT and KH domain constructs in BxPC‐3 cells after transfection. (M‐N) RNA immunoprecipitation (RIP) assays using IgG or HA antibodies to identify the IGF2BP2/3 domains binding to YAP1 mRNA.

**FIGURE 8:**
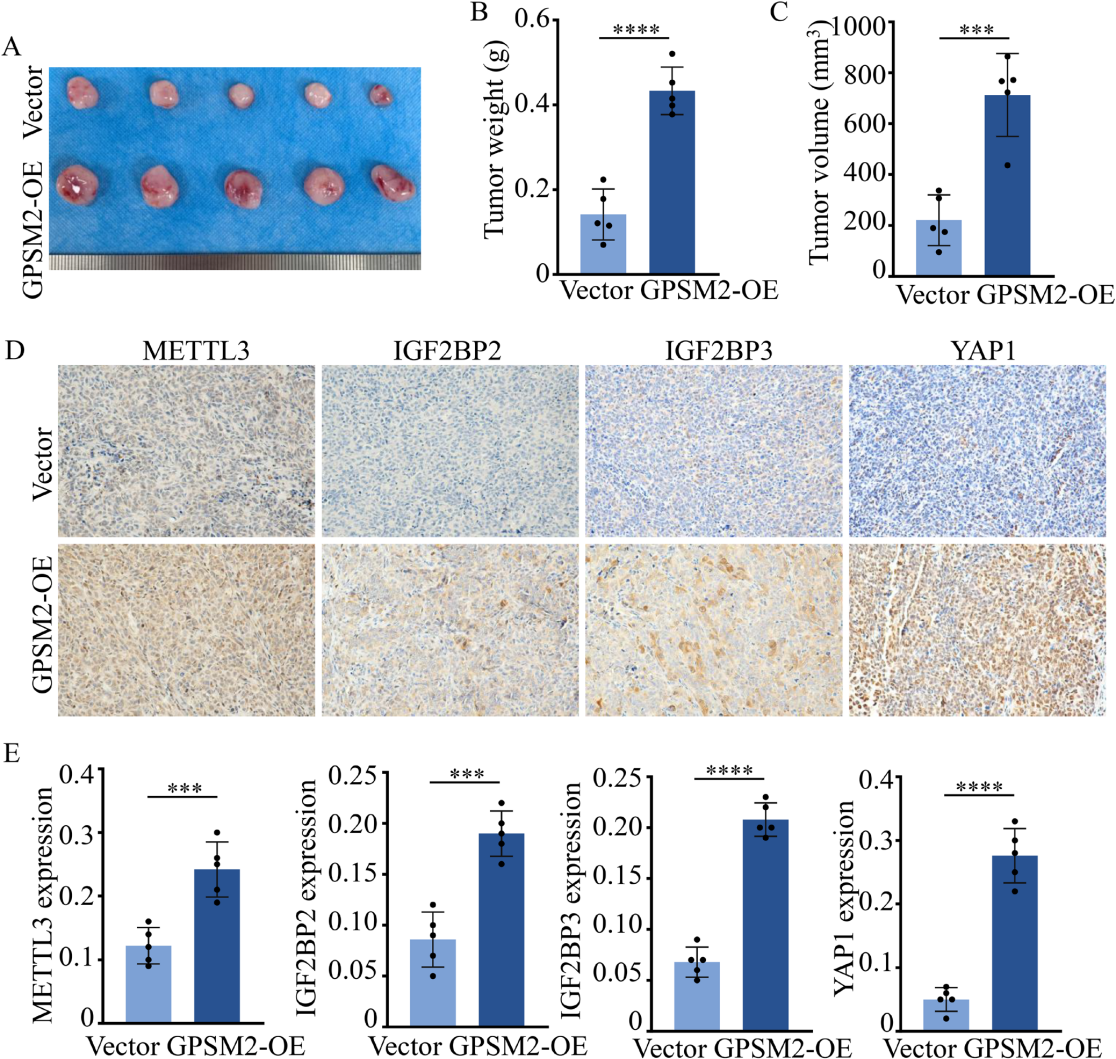
In vivo experiments were conducted to evaluate the proliferative capacity of pancreatic cancer cells. (A‐C) BxPC3 (Vector) and BxPC3 (GPSM2‐OE) cells were injected subcutaneously into athymic nude mice. Tumours were surgically excised and photographed (A), weighed (B) and their volumes were measured (C). ***p < 0.001. ****p < 0.0001. (D‐E) Immunohistochemical staining was performed to assess the expression of METTL3, IGF2BP2, IGF2BP3 and YAP1 in xenograft tumour tissues. ***p < 0.001. ****p < 0.0001.

M6A‐modified RNAs are predominantly recognised and functionally regulated by m6A reader proteins. To identify potential m6A readers involved in GPSM2‐mediated regulation of YAP1, we analysed TCGA pancreatic cancer data and found that high expression levels of IGF2BP2 and IGF2BP3 were associated with poorer overall survival in patients (Figure 7A). Additionally, both IGF2BP2 and IGF2BP3 expression levels were positively correlated with GPSM2 expression (Figure 7B,C). Overexpression of IGF2BP2 or IGF2BP3 in BxPC3(GPSM2) and PANC1(GPSM2) cells led to a significant upregulation of YAP1 protein expression (Figure 7D,G). Moreover, mRNA levels of YAP1 were also markedly increased upon overexpression of IGF2BP2 and IGF2BP3 in both cell lines (Figure 7H,I). These findings indicate that GPSM2 enhances YAP1 mRNA stability through IGF2BP2 and IGF2BP3. The IGF2BP family proteins contain two RNA recognition motif (RRM) domains and four K‐homology (KH) domains, which serve as RNA‐binding domains recognising m6A‐modified sites on mRNA [24]. Previous studies have reported that the KH domains of IGF2BPs primarily mediate mRNA stabilisation [21, 24]. To identify the specific domains of IGF2BP2 and IGF2BP3 responsible for binding to YAP1 mRNA, we constructed three HA‐tagged plasmids expressing different IGF2BP domain truncations (Figure 7J) and established stable pancreatic cancer cell lines expressing these constructs (Figure 7K,L). MeRIP‐qPCR assays performed in BxPC3 and PANC1 cells demonstrated significant enrichment of YAP1 mRNA by the KH3‐4 domains of IGF2BP2 and IGF2BP3 (Figure 7M,N). These results indicate that the KH3‐4 domains of IGF2BP2 and IGF2BP3 specifically recognise the m6A modification sites on YAP1 mRNA. Next, we further validated our conclusions using in vivo experiments. Xenograft tumour assays demonstrated that GPSM2 markedly promoted tumour growth in vivo (Figure 8A–C). Immunohistochemical analysis revealed that GPSM2 significantly enhanced the protein expression of METTL3, IGF2BP2, IGF2BP3 and YAP1 in tumour tissues (D‐E).

## 3 Discussion

Pancreatic cancer is one of the most common lethal malignancies. Despite rapid advancements in molecular diagnostics and therapeutic strategies in recent years, there remains a lack of clearly defined, effective targets for clinical treatment. Therefore, the identification of reliable biomarkers for pancreatic cancer is urgently needed. Xue et al. reported that GPSM2 is highly expressed in gastric cancer and promotes tumour cell proliferation and G1/S phase transition via the p53 pathway, thereby affecting patient prognosis [25]. Conversely, in lung cancer, GPSM2 knockdown has been shown to inhibit cancer cell proliferation both in vitro and in vivo [26]. In this study, through analysis of transcriptomic data from pancreatic cancer patients in the TCGA database, we identified GPSM2 as a gene significantly associated with overall survival in pancreatic cancer. Our in vitro experiments further confirmed that GPSM2 promotes colony formation and invasion of pancreatic cancer cells, consistent with findings by Zhou et al. [27]. However, we did not observe any significant association between GPSM2 expression and TNM stage or clinical grade in patients with pancreatic cancer, which may be attributed to the rapid progression of this malignancy. In future studies, this limitation may be addressed by enrolling a larger cohort of clinical samples to further evaluate the correlation between GPSM2 protein expression and clinicopathological staging and grading.

The canonical Hippo pathway transduces signals through a complex of serine/threonine kinases, mammalian Ste20‐like kinases 1/2 (Mst1/2) and the scaffold protein Salvador homologue 1 (Sav1). This complex phosphorylates and activates large tumour suppressor kinases 1/2 (Lats1/2) together with their regulatory proteins Mps One binder 1/2 (MOB1/2). Subsequently, Lats1/2 phosphorylates the transcriptional co‐activators YAP and TAZ, which can also serve as novel sensors of the mevalonate and glycolytic pathways [28, 29]. When the Hippo pathway is inactive, YAP localises to the nucleus, where it interacts with TEA domain DNA‐binding transcription factors (TEAD1‐4). Genes regulated by the YAP/TEAD complex encode proteins involved in numerous critical cellular processes and cross‐talk with other developmental pathways activated in pancreatic ductal adenocarcinoma (PDAC) [30]. YAP/TAZ further induces epithelial‐mesenchymal transition (EMT) and promotes a more undifferentiated, malignant cell state [29]. In this study, we found that GPSM2 enhances YAP1 protein expression, and mechanistic investigations revealed that GPSM2 promotes YAP1 protein levels by stabilising YAP1 mRNA.

N6‐methyladenosine (m6A) modification was first identified in the 1970s and has since attracted extensive attention as the most prevalent modification in eukaryotic messenger RNA (mRNA) and long non‐coding RNA (lncRNA) [31, 32]. M6A modification influences various biological processes by regulating mRNA splicing, translation and stability, thereby affecting cellular proliferation, invasion, metastasis and self‐renewal [33]. Recent studies have revealed that multiple m6A regulators are aberrantly expressed in pancreatic cancer and play critical roles in its pathogenesis and treatment [34].

The m6A modification on mRNA is catalysed by a multicomponent m6A methyltransferase complex (MTC), which consists of the core heterodimer formed by methyltransferase‐like 3 (METTL3) and methyltransferase‐like 14 (METTL14), along with other regulatory subunits including KIAA1429, WTAP, ZC3H13 and RBM15/RBM15B. Among these components, METTL3 is the sole subunit capable of binding the methyl donor S‐adenosylmethionine and catalysing the methyl transfer, playing a pivotal role in m6A modification [35]. Tang et al. reported that METTL3 is overexpressed in pancreatic cancer and promotes cancer growth and metastasis by enhancing the mRNA stability of E2F5 through m6A methylation [36]. Chen et al. demonstrated that the METTL3‐YTHDF2 axis mediates m6A modification that suppresses ID2 mRNA stability; ID2 regulates stemness factors NANOG and SOX2 via the PI3K‐AKT pathway to support pancreatic cancer growth and stemness maintenance [37]. In the present study, we found that GPSM2 upregulates METTL3 expression, thereby inducing m6A modification of YAP1 mRNA. Interestingly, although both METTL14 and WTAP were positively correlated with GPSM2, neither appeared to be directly regulated by GPSM2, which may be attributable to the fact that our analysis was based on transcriptomic data. Furthermore, the m6A reader proteins IGF2BP2 and IGF2BP3 recognise m6A sites on YAP1 mRNA through their KH3‐4 domains, enhancing YAP1 mRNA stability and consequently upregulating YAP1 protein expression, which promotes pancreatic cancer cell proliferation and invasion.

In summary, we demonstrate that GPSM2 promotes METTL3‐mediated m6A modification of YAP1 mRNA, with IGF2BP2 and IGF2BP3 serving as the primary m6A readers recognising these modifications. This interaction enhances the stability of YAP1 mRNA, thereby upregulating YAP1 protein expression, which in turn facilitates pancreatic cancer cell proliferation and invasion. These novel findings provide valuable insights into the pathogenic mechanisms of pancreatic ductal adenocarcinoma (PDAC) and offer potential avenues for targeted therapeutic development. Given that the present study did not comprehensively investigate the regulatory mechanism between GPSM2 and METTL3, nor the mechanistic dependency between GPSM2 and YAP1, future studies will further elucidate these interactions through independent in vivo and in vitro experiments.

## 4 Materials and Methods

### 4.1 Plasmids, Reagents and Antibodies

Plasmids encoding GPSM2 and METTL3 were acquired from Addgene (Cambridge, MA, USA). siRNA GPSM2 (sc106999), siRNA METTL3 (sc‐92,172), siRNA METTL14 (sc‐89,054) and siRNA WTAP (sc‐63,224) plasmids were obtained from Santa Cruz Biotechnology (Santa Cruz, CA, USA). Plasmids encoding IGF2BP2 and IGF2BP3 were acquired from MiaoLing (Wuhan, China). Plasmid preparation was conducted utilising the Plasmid Preparation/Extraction Maxi kit supplied by QIAGEN (Valencia, CA, USA). Verteporfin was procured from Med Chem Express (MCE, China). Antibodies against GPSM2 (26798–1), METTL3 (15073–1), METTL14 (26158–1), WTAP (60188–1), IGF2BP2 (11601–1), IGF2BP3 (14642–1), YAP1 (13584–1), GAPDH (60004–1), β‐actin (66009–1) and α‐Tubulin (66031–1) were sourced from Protein Tech Biotechnology (Protein Tech, China). The forward primer of IGF2BP2 KH1‐4 domain truncation: 5′‐ATG GATTTCCCGCTGCGGATC‐3′ and reverse primer 5′‐CTGTGCAGTCTGGCTAG −3′; the forward primer of IGF2BP2 KH1‐2 domain truncation: 5′‐ATGGATTTCCCGCTGCGGATC‐3′ and reverse primer 5′‐AATCTCTATCTCAGCACT −3′; the forward primer of IGF2BP2 KH3‐4 domain truncation: 5′‐ATGCAGGAGATTGTGAAT‐3′ and reverse primer 5′‐CTGTGCAGTCTGGCTAG −3′. The forward primer of IGF2BP3 KH1‐4 domain truncation: 5′‐ATGGATTTGCCTCTGCGCCTG‐3′ and reverse primer 5′‐AATTTTTCTCTGGGCAAC −3′; the forward primer of IGF2BP3 KH1‐2 domain truncation: 5′‐ATGGATTTGCCTCTGCGCCTG‐3′ and reverse primer 5′‐GATCTCCTCCTCAGCTTT −3′; the forward primer of IGF2BP3 KH3‐4 domain truncation: 5′‐ATGACGGAGACTGTTCAT‐3′ and reverse primer 5′‐AATTTTTCTCTGGGCAAC −3′. The sequence of sgRNA targeting *Yap1* was as follows: gRNA: ACGACCTGGTGACCCGCCGG.

### 4.2 Cell Culture and Transfection

Human pancreatic cancer cell lines BxPC3 and PANC1 were obtained from the American Type Culture Collection (Manassas, VA, USA). BxPC3 and PANC1 were cultured in Dulbecco's modified Eagle's medium (Gibco, Carlsbad, CA, USA) and incubated at 37°C in a 5% CO_2_ humidified atmosphere. All these cell lines were routinely authenticated for purity and were infection‐free. Stable transfection was performed using PolyJet DNA In Vitro Transfection Reagent, consistent with the manufacturer's guidelines.

### 4.3 Quantitative RT‐PCR for mRNA Analysis

Total RNA was isolated utilising TRIzol reagent (Invitrogen, USA) and reverse transcribed into cDNA. Specific primer pairs were deployed for the amplification of targeted genes as delineated in the corresponding figures and as previously described [38]. The primers utilised in this study were listed as follows: for YAP1, Forward‐5′‐TAGCCCTGCGTAGCCAGTTA‐3′ and Reverse‐5′‐TCATGCTTAGTCCACTGTCTGT‐3′; for GAPDH, Forward‐5′‐GGAGCGAGATCCCTCCAAAAT‐3′ and Reverse‐5′‐GGCTGTTGTCATACTTCTCATGG −3′.

### 4.4 Methylated RNA Immunoprecipitation (MeRIP) Assay

MeRIP assays were conducted with Magna MeRIP m6A Kit (#17–10,499, Merck Millipore). Purified mRNA was digested by DNase I and then fragmented into 100 nt using RNA fragmentation reagent and incubated at 94°C. After fragmenting, the stop buffer was added, following which standard ethanol precipitation was performed and collected. The anti‐m6A antibody for 12 μg was pre‐incubated with 50 μL beads in IP buffer (150 mM NaCl, 0.1% NP‐40, 10 mM Tris–HCl, pH 7.4) at room temperature for 1 h. Next, 6 μg of fragment mRNAs was added to the antibody‐beads mixture and incubated at 4°C for 4 h on a rotator. After adequate washing, the immunoprecipitated mixture was digested using a high concentration of proteinase K, and the bound RNAs were extracted using the phenol‐chloroform method and ethanol precipitation and were used for qPCR analysis.

### 4.5 RNA Immunoprecipitation (RIP) Assay

The RNA‐IP procedure was carried out in accordance with methodologies previously reported [39]. The cells were suspended and broken down in RIP lysis solution. The cell lysates were mixed with RIP immunoprecipitation buffer that had magnetic beads attached to anti IGF2BP2/3 antibody and control IgG antibody. This mixture was then incubated overnight at a temperature of 4°C. Following incubation with Proteinase K, the RNA that was immunoprecipitated was eluted, isolated and measured using qRT‐PCR.

### 4.6 Western Blot Analysis

This was conducted in strict adherence to previously established protocols. Protein samples were processed and subjected to Western blotting using specified antibodies [40]. Visualisation and capture of the resultant immuno‐reactive bands were facilitated through scanning via a Typhoon FLA 7000 imager (Pittsburgh, PA, USA) and analysed using ImageJ Software.

### 4.7 RNA Decay Assay

The cells were seeded into six‐well plates. Then, actinomycin D (Selleck) was added to cells with a final concentration of 10 μg/mL. After incubation for 0, 2, 4 and 8 h, cells were successively harvested to extract the total RNAs for subsequent RT‐qPCR assays.

### 4.8 RNA m6A Quantification

The m6A modification level of total RNA was examined via EpiQuik m6A RNA Methylation Quantification Kit (P‐9005; Epigentek Group Inc., Farmingdale, NY, USA) according to the instructions. Briefly, 200 ng RNA accompanied by the m6A standard were coated on assay wells, followed by capture antibody solution and detection antibody solution. The m6A levels were quantified colorimetrically by reading the absorbance of each well at a wavelength of 450 nm (OD450), and then calculations were performed based on the standard curve.

### 4.9 Cell Migration and Invasion Assays

In vitro migration and invasion assays were performed using Transwell chambers and Matrigel‐coated chambers, respectively, following the protocols provided by the manufacturer (BD Biosciences, Bedford, MA), consistent with previously described methods [41]. Quantification of migrated and invasive cells was achieved using ImageJ software (NIH, Bethesda, MD, USA), with results being representative of data collected from three independent experiments.

### 4.10 Colony Formation Assay

Approximately 1000 cells were plated into six‐well plates and cultured for 1 day. The fresh medium was added to the cells and further incubated for 2 weeks before the cells were fixed with 4% formaldehyde and stained with 0.5% crystal violet. The number of colonies was then counted under a microscope and analysed using ImageJ Software.

### 4.11 Xenograft Tumorigenic Model

The xenograft tumour experiments were conducted at the Wenzhou Institute UCAS in accordance with the protocol approved by the Experimental Animal Care Committee of Wenzhou Institute UCAS (licence no. SYXK, Zhejiang 2021–0040). 5‐ to 6‐week‐old female athymic nude mice were randomly divided into two designated groups and subcutaneously injected with various BxPC3 cell transfectants (2 × 10^6^ cells suspended in 150 μL). 4 weeks post‐injection, euthanasia was performed and tumours were surgically removed, photographed and weighed.

### 4.12 Immunohistochemistry Staining (IHC)

The mice xenograft tumour slides were stained using the M&R HRP/DAB Detection IHC Kit (Cat. HC301‐01, Vazyme, Nanjing, China). The integral optical density (IOD) per area was quantified to assess protein expression levels by utilising Image Pro Plus 6.0 (Media Cybernetics, MD, USA) [42]. The IHC‐stained slides were evaluated at 200× magnification, and at least five fields of view per slide were analysed to calculate the optical density based on characteristic images.

### 4.13 Immunoprecipitation

Cells are washed with cold PBS and lysed in cell lysis buffer (9803, CST) containing complete protein inhibitor cocktail (04693116001, Roche). The cell lysates were centrifuged at 14,000 × *g* for 10 min at 4°C. The protein concentration in the whole cell lysate was detected by BCA (23,227, Thermo Fisher) and the samples were divided into Input and IP. The IP samples were incubated with the indicated GPSM2 antibodies and protein A/G agarose beads (Santa Cruz) at 4°C for immunoprecipitation. Similarly, tag magnetic beads (anti‐HA‐tag: M180‐11; Anti‐DDDDK‐tag: M185‐11R, MBL) were incubated at 4°C for co‐immunoprecipitation. Protein complexes were resuspended with boiling buffer and detected by western blotting.

### 4.14 Bioinformatic Analyses

Transcriptome RNA‐seq data of 183 pancreatic cancer cases (normal samples, 5 cases; tumour samples, 178 cases) and the corresponding clinical data were downloaded from the TCGA database (https://portal.gdc.cancer.gov/). To improve the robustness of tumour–normal comparisons, normal pancreatic tissue transcriptome data were additionally obtained from the GTEx database. For differential expression analysis, TCGA‐PAAD tumour samples were integrated with GTEx normal pancreatic tissues and the available adjacent normal pancreatic samples from TCGA. After normalisation and batch correction, gene expression values were used for downstream analysis. Differential gene expression analysis was performed using the limma package in R, and DEGs were generated. DEGs with a fold change larger than 1 after transformation of log2 (high‐score group/low‐score group) and false discovery rate (FDR) < 0.05 were considered significant. Heatmaps of DEGs were produced using the R language with the package pheatmap. Volcano plot of DEGs was produced by the R language with the package ggscatter. GO and KEGG enrichment analyses using DEGs were performed with the R language with the aid of packages clusterProfiler, enrichplot and ggplot2. Only terms with both *p* and *q* values of < 0.05 were considered significantly enriched. R language, loaded with the package survival and survminer, was applied for the survival analysis. The Kaplan–Meier method was used to plot the survival curve, and log rank as the statistical significance test; *p* < 0.05 was considered significant. R language loaded with the package survival was used for univariate COX regression. The top 10 genes ordered by HR value from largest to smallest in univariate COX were shown in the plot.

### 4.15 Statistical Analysis

All statistical analyses were performed using GraphPad Prism and R software. Data are presented as the mean ± SD from at least three independent experiments. Comparisons between two groups were performed using an unpaired two‐tailed Student's *t*‐test. Comparisons among multiple groups were performed using one‐way ANOVA followed by Tukey's multiple‐comparison test. Survival curves were analysed using the Kaplan–Meier method with the log‐rank test. Univariate Cox regression analysis was used to assess the association between gene expression and overall survival. A *p* value < 0.05 was considered statistically significant.

## Author Contributions

**Zixuan Shen:** data curation, formal analysis. **Xuelei Liu:** writing – review and editing, data curation, formal analysis, software, supervision, project administration. **Xiaoan Hu:** formal analysis, data curation. **Jiajun Xiu:** writing – original draft, software. **Miaomiao Li:** formal analysis, funding acquisition. **Li Qiao:** data curation, validation. **Yinghui Zhang:** writing – review and editing, funding acquisition, project administration. **Rui Yang:** software. **Hairu Zhang:** formal analysis. **Zhe Dong:** methodology, formal analysis.

## Funding

This work was supported by the Hospital Fund of the First Hospital of Lanzhou University (No. ldyyyn2022‐86) and the Lanzhou Science and Technology Planning Project (2023‐ZD‐94).

## Consent

All authors have agreed to publish this manuscript.

## Conflicts of Interest

The authors declare no conflicts of interest.

## Data Availability

The data that support the findings of this study are available from the corresponding author upon reasonable request.
